# Clinic for Multimorbidity: An Innovative Approach to Integrate General Practice and Specialized Health Care Services

**DOI:** 10.5334/ijic.7015

**Published:** 2023-06-12

**Authors:** Cathrine Bell, Peter Vedsted, Dorte Gunver Adsersen Kraus, Ulrich Fredberg, Linda Jeffery, Marianne Bjørn Dahlgaard, Rikke Aarhus, Charlotte Weiling Appel

**Affiliations:** 1 Diagnostic Centre, University Research Clinic for Innovative Patient Pathways, Silkeborg Regional Hospital, Silkeborg, Denmark; 2 Research Unit for General Practice, Faculty of Health, Aarhus University, Denmark; 3 Department of Rheumatology, Odense University Hospital, Odense, Denmark; 4 Hospital Pharmacy, Central Jutland Region, Denmark

**Keywords:** integrated care, multimorbidity, outpatient clinic, primary care, polypharmacy, chronic disease

## Abstract

**Introduction:**

Caring for patients with multimorbidity in general practice is increasing in amount and complexity. To integrate care for patients with multimorbidity and to support general practitioners (GPs), the Clinic for Multimorbidity (CM) was established in 2012 at Silkeborg Regional Hospital, Denmark. This case study aims to describe the CM and the patients seen in it.

**Results:**

CM is an outpatient clinic that offers a comprehensive one-day assessment of the patient’s complete health status and medication. GPs can refer patients with complex multimorbidity (≥2 chronic conditions). It involves collaboration across medical specialties and healthcare professions. The assessment is completed with a multidisciplinary conference and recommendation.

In all, 141 patients were referred to the CM between May 2012 and November 2017. The median age was 70 years, 80% had more than five diagnoses, and in median patients had a usage of 11 drugs (IQI, 7–15). Physical and mental health was reported low (SF-12 score: 26 and 42). In median four specialties were involved and 4 examinations (IQI, 3–5) conducted.

**Conclusion:**

The CM offers innovative care by bridging and exceeding conventional boundaries of disciplines, professions, organizations, and primary and specialized care. The patients represented a very complex group, requiring many examinations and involvement of several specialists.

## Introduction

Management of multimorbidity is one of the greatest health-related challenges facing patients, health professionals, and society in general. The term multimorbidity is commonly defined as the concurrent presence of two or more chronic conditions in an individual [1,2]. Multimorbidity is estimated to be present in 29% of the general adult population [3]. The rising prevalence of multimorbidity, in a population of steadily increasing life expectancy [4,5,6], poses considerable challenges for the general practitioner (GP) [7]. Often these patients need frequent consultations, and the time consumption and burden of their contacts tend to rise with the number of chronic conditions [8,9]. Chronic conditions tend to compound and interact, thereby producing healthcare issues and drug regimens of high complexity. For the main part, patients with multimorbidity are treated in the primary care setting, and account for 53% of GP consultations and 79% of prescriptions [10] with the GP is assigned to the coordinating and gatekeeping function for the patient care. However, GPs are challenged by insufficient collaboration between healthcare professionals involved in the treatment [7,8,11,12,13] and by healthcare services and guidelines that usually are built around treating single diseases [7,14,15]. In hospitals, a focus on single-diseases and a specialized healthcare system has led to fragmented and less efficient care for patients with complex multimorbidity, leaving the GP in difficulty navigating the care [8,16,17,18].

The societal challenge and financial burden of increasing numbers of patients with multimorbidity along with the consequences of work burden and fragmented care entail a growing demand for multidisciplinary care tailored to accommodate the individual’s health and needs [19]. The GP can facilitate a more targeted effort and prioritization of healthcare resources, based on the patients’ assigned risk level, which enables a graduated effort to be launched for selected chronic conditions [20]. This is called risk stratification and is considered in line with the Chronic Care Model [20], which aims to improve care for chronic conditions in a multidimensional manner. The Chronic Care Model involves six components of healthcare delivery that encourage high-quality chronic disease care: the community, the health system, self-management support, delivery system design, decision support and clinical information systems [21]. According to the Chronic Care Model, it requires a planned and coordinated effort between all involved in the patient’s care across sectors to further a proactive, planned and population-based care, yet person-centered [21]. Integrated care programs are urged to respond to the need of improved collaboration across sectors and disciplines, as proposed in the Chronic Care Model and solutions for person-centered care is called for [22]. However, few interventions exist for improving outcomes in patients with complex multimorbidity and polypharmacy [23,24,25,26,27,28,29,30,31]. One example is the Clinic for Multimorbidity (CM) established in 2012 at Silkeborg Regional Hospital, Denmark [32]. This case study aims to describe the CM and its patients in the attempt to promote integrated care for people with complex multimorbidity and polypharmacy by supporting the GP in care management.

## Context

The Danish health care system is financed through taxes with universal access to general practice, hospitals, and some allied services [14]. GPs act as gatekeepers to the specialized health sector and 99% of Danish citizens are listed with a general practice as their primary point of contact for medical advice. While most chronically ill patients have regular visits at their GP, the more complex cases are also followed in the specialized healthcare system at hospitals in outpatient clinics.

The CM was established at Diagnostic Centre, Silkeborg Regional Hospital, a part of the Regional Hospital Central Jutland, in Central Denmark Region. The CM at has a catchment area of approximately 189,000 residents from 18 years of age. At the Diagnostic Centre, all internal medical specialties are represented (pulmonology, cardiology, endocrinology, rheumatology, gastroenterology and hepatology, geriatrics, infectious diseases, hematology, and nephrology). In addition, the CM has easy and fast access to paraclinical examinations including radiology and is situated in proximity to municipality-driven psychiatry, which facilitates collaboration.

## Description of the Care Practice

### Development

The CM was developed in 2012, co-created by clinicians, management, and facilitating officers from Silkeborg Regional Hospital, together with local GPs. The CM builds on elements of the Chronic Care Model and was founded as user-driven innovation; clinical experience, along with an expressed need from the healthcare professionals collaborating across the primary and secondary sector, and research underpinning the need for integration of existing services. The assumptions behind setting up this organization were, that a joint treatment plan achieved from intersectoral and interdisciplinary collaboration with a person-centered approach would optimize the quality of care and support GPs in care management of complex patients with multimorbidity.

### The Clinic for Multimorbidity

People with complex health problems and suffering from at least two chronic conditions (including psychiatric illnesses) can be referred to the CM by their GP. When a referral is accepted, a personal consultant initially collects all relevant information from the patient files and writes a chronological summary of the patient’s medical history and previous treatments. The consultant requests relevant tests needed prior to the visit at the clinic, and a pathway coordinator (nurse or secretary) organizes these tests to all be conducted in one day closely before the primary consultation. The personal consultant is a medical doctor and acts as a generalist across the spectrum of medical conditions.

At the primary consultation to the CM, the coordinator welcomes the patient and measures vital values. The medication is reviewed with the patient by a pharmacist, followed by an assessment from a physiotherapist and an occupational therapist. The pharmacist and therapists report any anomalies/focus areas to the personal consultant before the consultation with the patient takes place. The personal consultant then has a consultation with the patient, focusing on the patient’s individual needs and concerns. After this, and on the same day, a multidisciplinary conference takes place, with the participation of the personal consultant, other relevant medical specialists from the Diagnostic Centre, the pharmacist, therapists, and the pathway coordinator, at which a joint and comprehensive treatment plan for the patient is proposed. Conference participants are selected by the personal consultant and organized by the pathway coordinators. It is possible for the GP to participate by videoconference. The patient, who does not attend the conference, is offered a meal and a place to rest, while awaiting the outcome of the conference. At the conference, a short summary of the patient’s medical history, medication, and current issues are presented. Treatment options are discussed and when agreed upon by all participants, a proposal for a final treatment plan is made. This can include alterations in current medication, suggestions on discontinuation or tapering, establishing necessary homecare and a rehabilitation plan. After the conference, the personal consultant presents the patient with a proposed treatment plan. The treatment plan is agreed upon, then sent to the GP and stored in the hospital’s electronic records. This can end the intervention or when relevant, there may be follow-up visits. Care following the CM is placed back with the GP, who is now provided with specialist suggestions in a unified treatment plan.

Thus, the CM integrates primary care with specialized care and offers a holistic overview of patients with complex multimorbidity and their problems to support GPs responsible for the care and focusses specifically on the patient’s individual needs.

### Data description

We included descriptive data on the 141 patients aged ≥18 years, referred to the CM at Diagnostic Centre, Silkeborg Regional Hospital between May 2012 and November 2017.

The personal consultant or pathway coordinator had documented; patients seen in the CM, age and gender, Body Mass Index, diagnoses presented using International Classification of Diseases, 10^th^ revision, referring units (GP or hospital), clinical examinations and tests, whether video conference was involved (yes/no), medical specialties involved, medication usage based on the Anatomical Therapeutic Classification System, tobacco usage (smoker, on occasions, former and never), and alcohol consumption (0–1, 2–10 and >10).

Information on patients with follow-up visits three months after visiting the CM were retrieved from the patient administrative system, which keeps record of all hospitalizations and outpatient treatments in Central Region Denmark.

Questionnaires, which included data on working status, educational level, civil status, and physical and mental wellbeing, were filled out by the patients at the CM. Working status was grouped as working (full/part-time and sick leave), not working, on pension (including early retirement), undergoing education, or on social benefit. Educational level was grouped into <10, 10–15, and >15 years of education according to the International Standard Classification of Education, 11^th^ revision. Civil status was reported dichotomized as living with partner/living alone. To assess well-being, the 12-item Short Form Health Survey (SF-12) instrument was used [33]. The SF-12 is comprised of eight subscales. These were summarized into a physical component score (PCS) and a mental component score (MCS), in accordance with the guidelines for the SF-12 instrument. Both scores range between 0 and 100, with a higher score indicating better health.

### Statistical analysis

Descriptive statistics by counts with percentages and medians with interquartile intervals were used. All analyses were conducted in Stata version 17.

## Results

In total, 141 patients were assessed at the CM. Females constituted 60% and the median age was 70 years (IQI, 63–77). Non-workers constituted 86% and 45% lived alone. Hypertension (51%), diabetes type-2 (35%), and atrial fibrillation/flutter (30%) were the most frequent diagnoses and 80% of the patients had more than five diagnoses. In median, patients had a usage of 11 drugs (IQI, 7–15) and the most frequently used drugs were related to the cardiovascular system (86%), the digestive organs and metabolic system (69%), and the nerve system (66%). General health, physical and mental wellbeing was reported low, scoring 25, 26, and 42 on SF-12, respectively (Table 1).

**Table 1:** Characteristics of the 141 patients, who entered the Clinic for Multimorbidity at Silkeborg Regional Hospital between May 2012 to November 2017.


	n		%^1^

**Total**	141		(100)

**Gender**			

Male	57		40.4

Female	84		59.6

**Age** (years)	Median = 70		(IQI, 63–77)

<60	25		17.7

60–69	43		30.5

70–79	48		34.0

≥80	25		17.7

**Working status**			

Working	11		7.8

Pension	116		85.8

Social benefits	3		2.1

Studying	2		1.4

Missing	9		6.4

**Educational level** ^1^			

<10 years	24		17.0

10–15 years	54		38.3

>15 years	23		16.3

Missing	40		28.4

**Civil status**			

Living with partner	76		54.9

Living alone	61		44.5

Missing	4		2.8

**Body Mass Index**	Median = 28.2		(IQI, 24–32)

**Number of diagnoses**	Median = 9		(IQI, 7–11)

<5	25		17.7

5–9	70		49.7

≥10	39		29.1

Missing	7		2.8

Diagnoses, most frequent*			

Hypertension [DI10]	72		51.1

Diabetes, type 2 [DE11]	50		35.5

Atrial fibrillation and flutter [DI148]	42		29.8

Chronic obstructive lung disease [DJ44]	42		29.8

Dyslipidaemia [DE78]	37		26.2

Chronic ischemic heart disease [DI25]	35		24.8

**Drug usage**^3^ **(N = 128)**	Median = 11		(IQI, 7–15)

*Drugs related to*	*N of drugs*	*N with drugs*	

Digestive organs and metabolism	219	96	68.7

Blood and blood-forming organs	108	81	57.5

Cardiovascular system	398	121	85.8

Dermatological system	24	24	17.0

Urogenital system and gender hormones	40	32	22.7

Hormones, systematic use	76	61	43.3

Infectious diseases, systematic use	112	65	46.1

Antineoplastic and immunomodulatory drugs	8	8	5.7

Musculoskeletal system	71	63	44.7

Nerve system^4^	288	93	66.0

Antiparasitic products, insecticides, and repellents	6	6	4.3

Respiratory system	174	63	44.7

Sensory organs	30	21	14.9

Others	35	35	24.8

**Tobacco usage**			

Smoker	26		18.4

On occasions	3		2.1

Former smoker	58		41.3

Never	31		22.0

Missing	8		5.7

**Alcohol consumption, daily**			

0–1	51		36.2

2–10	14		9.9

>10	10		7.1

Missing	66		46.8

**Health status, scored by SF-12**	78		55.3

Physical well-being	Median = 25.7		(IQI, 19.6–32.4)

Mental well-being	Median = 42.3		(IQI, 33.3–48.9)


^1^ May not add up to 100% due to missing values or rounding. ^2^ Based on most frequent diseases by International Classification of Diseases, 10^th^ revision. ^3^ As grouped in the Anatomical Therapeutic Chemical Classification System (ATC codes). ^4^ Including pain medication. IQI: Interquartile Interval.

Patients waited in median 31 days (IQI, 20–50) from referral to entrance to the CM. In 83% of cases, a GP had referred the patient (Table 3). Before the primary consultation at the CM, a median of four examinations (IQI, 3–5) were conducted, the most frequent being electrocardiograms (79%), urine analyses (62%), and lung function examination (31%) (Table 2). Blood sampling was requested in 96% of cases.

**Table 2:** Examinations related to the consultation in the Clinic for Multimorbidity for 141 patients who entered between May 2012 and November 2017.


	n	%^1^

**Examinations**	Median 4	(IQI, 3–5)

Blood samples	135	95.7

Blood sugar testing × 4 within a week	1	0.7

Stool samples	7	5.0

Synacthen test	5	3.6

Lung function examination	43	30.5

Electrocardiogram	111	78.7

Holter monitoring	8	5.7

Echocardiogram	41	29.1

Ultrasound or X-ray	20	14.2

Bone density scan, Dexa	7	5.0

Urine analyses	87	61.7

Other^2^	4	2.8


^1^ May not add up to 100% due to missing values or rounding. ^2^ Others include one examination for each: orthostatic blood pressure measurement, biothesiometry, helicobacter breath test, and schirmer’s test. IQI: Interquartile interval.

**Table 3:** Characteristics of involvement before, during and after patients had entered the Clinic for Multimorbidity.


		n	%^1^

**N with examinations, 3 months after first visit2**		90	63.8

**N with visits in the Clinic, 3 months follow up**		69	48.9

**Referring unit**			

GP		117	83.0

Hospital		24	17.0

**Video conference involved**			

Yes		11	8

No		126	89.4

Missing		4	3.0

**Number of specialties involved**	Median 5 (IQI, 3–5)		

Endocrinology		110	78.0

Gastroenterology		50	35.5

Geriatric		51	36.2

Hematologic		4	2.8

Infection medicine		4	2.8

Cardiology		102	72.3

Pulmonology		72	51.1

Nephrology		21	14.9

Psychiatry		25	17.7

Radiology		1	0.7

Rheumatology		95	67.4

Occupational therapy		19	13.5

Pharmacist		46	32.6

Physiotherapy		23	16.3

Other		4	2.8


^1^ May not add up to 100% due to rounding. GP: General practitioner. IQI: Interquartile interval. ^2^ Initiated by the Clinic for Multimorbidity. GP: General practitioner.

In median, four medical specialties (IQI, 3–5) were represented at the conference; endocrinology, cardiology, rheumatology, and pulmonology being the most frequent specialties attending. Video conference was established with the GPs in 8% of cases. Patients with examinations at three months follow-up constituted 63.8%, and patients visiting the CM at three months follow-up made up 48.9% (Table 3).

## Discussion

### In summary

The CM involves health care collaboration, knowledge sharing and shared treatment planning. It was the first of its kind in Denmark and creates an innovative patient pathway for people with multiple diseases. It integrates different care professionals and links primary and hospital sectors. The 141 patients visiting the CM during the investigation period were characterized by having a high number of chronic conditions, a high medicine usage, and a low self-reported physical well-being. Overall, they represented a very complex group of patients with multimorbidity, requiring a wide range of examinations and involvement of several specialists.

### Comparison with existing literature

Research on interventions aiming to support the GPs in care management of patients with multimorbidity are sparce [23,24,25,34]. In an umbrella review by Damery et al., one of the main findings on integrated care effective interventions to reduce hospital activity concerned multidisciplinary teams [35]. Another umbrella review by Flanagan et al. presented promising evidence of effectiveness in case management interventions to improve the patient’s quality of life [36]. Furthermore, across several European countries there is consensus that key aspects for integrated care involve a holistic view of the patient, continuity of care in the form of single contact points and continuous communication, alignment of services, good relationships, trust, and patient involvement [37]. The CM attempts to incorporate or facilitate these aspects while the role as gatekeeper and case manager remains with the GP. Since the establishment of the CM in 2012 more outpatient clinics and care pathways with provision of integrated care solutions for patients with multimorbidity have emerged in hospitals in Denmark, which to some extent, resemble the CM [25,38]. Setting up an organization with multidisciplinary efforts require much coordination [38] and the inclusion of patients in the CM is reliant on GP awareness for an add-on and collaborative effort of specialties. Caring for and coordinating care for patients with complex multimorbidity is a demanding task [12,39,40]. One study found that the GP’s time consumption and the burden of contacts tend to rise with the number of chronic conditions [8], another study found that service provision for chronic care vary considerably across general practices [41]. The CM had a low inclusion as only 141 patients underwent the intervention during the time of investigation. We question whether the intervention was sufficiently implemented, as GP awareness (and finding the offer beneficial) is the cornerstone for referral to the CM. A qualitative study by Nissen et al. found that GPs, who referred to the CM, experienced difficulties in determining the suitable time for referral, and consequently referred few and not necessarily the most in need [13].

Our results show that patients seen at the CM had a high number of diagnoses and a high level of polypharmacy, requiring involvement of several healthcare professionals. Compared to a Danish national health survey with four or more chronic conditions [42], CM patients experienced lower physical well-being (score 26 versus 38), underpinning that these were physical fragile patients. Providing the GPs with a referral option for complex patients with multimorbidity was the intention with the CM, and risk stratification may be effective in locating patients with complexities suited for referral. Other common characteristics seen in the CM were female gender, high age, no labor market attachment, and a high number of patients with conditions or drugs related to the circulatory system. These resemble characteristics in former intervention studies aimed at people with multimorbidity [38,43,44].

When dealing with patients with multimorbidity, GP care is commonly influenced by insufficient communication and coordination, unclear divisions of roles, and responsibilities across sectors [13]. Care transition from the GP to the CM and vice versa may also be prone to these challenges. Nissen et al. also found that the CM needs clarity on intervention elements in terms of “who does what”, for its users to find it lucrative. Care suggestions from the CM are provided to the GP, who are left with realizing these suggestions, which may include difficultly obtainable goals [13]. Follow-up visits as part of a personal health care plan took place but was not standard. These elements may affect GP referral and in part explain the low inclusion of patients in the CM.

### Implications

The CM was an attempt to create a more efficient trajectory for patients with complex multimorbidity and in need of case management. The CM was an intervention grounded in the Chronic Care Model and the pyramid of risk stratification [20], where the most challenged patients and their GPs are provided with a supportive solution. More information on social determinants of health would expand our description and had social determinants been incorporated in the care model, it might have facilitated a broader approach to care [45]. Still, the CM comprises many of the elements from the Chronic Care Model for fostering productive chronic care delivery: integrating specialists for decision support and case management for complex patient care, sharing information and responsibilities, making action plans and goal setting, and promoting safe high-quality care across specialties and sectorial borders. The focus was to support GPs in being lead on the disease course and to support the decision-making on treatment by offering a review of the patients’ complete disease status including advice on optimizing the treatment plan, without having to send the patient for serial examinations by different medical specialists. Although, the GP serves as gatekeeper to specialized care in this context, the CM may still be relevant to systems without this initial point of contact. In this case, it would be relevant to consider how the patient is referred and how responsibility is placed following the intervention. The CM has the advantage of being a clinic with possibility to coordinate collaboration and knowledge-sharing of specialists for already diagnosed patients and ought not to be considered as a diagnostic clinic.

### Strengths and limitations

The data is collected prospectively over a long period and provides a detailed description of the practice implemented. As a descriptive case study, it lacks results on effectiveness and costs. Which decisions were made at the conference and how often changes were made in the medical care plan lacks description. The CM was not established as a research intervention, instead the intervention was formed from an identified need through clinical experience and former literature and theory, and an inclination to create an integrated solution. In its simplicity, we present a case description and believe that elements of the CM can be an inspiration to future care organization for patients with multimorbidity. Practicalities of organizing the clinic are manageable, however, this demands close collaboration between diverse healthcare professionals and a general prioritizing of a resource-consuming intervention. Research is needed on assessing costs and effectiveness of the CM. Research is also needed on the care outcome of the CM intervention and how this affects patient care following the intervention.

## Conclusion

The study presents a description of the CM, which offers innovate care by bridging and by exceeding conventional boundaries of disciplines, professions, organizations, and primary and specialized care. The CM may benefit patients by facilitating integrated care, however, it may be regarded as a resource-consuming intervention judging by its organization. More research is needed describing the delivery and form of the multidisciplinary care plans and how this is put into practice and on effectiveness and costs related to the intervention.

## Figures and Tables

**Table:** 

CM	Clinic for Multimorbidity
GP	General Practitioner
IQI	Interquartile Interval
MCS	Mental Component Score
PAS	Patient Administrative System
PCS	Physical Component Score
SF-12	Short Form Health Survey, 12 items

## References

[1] Johnston MC, Crilly M, Black C, Prescott GJ, Mercer SW. Defining and measuring multimorbidity: a systematic review of systematic reviews. Eur J Public Health. 2019; 29(1): 182–9. DOI: 10.1093/eurpub/cky098 29878097

[2] Van den Akker M, Buntinx F, Knottnerus JA. Comorbidity or multimorbidity. Eur J Gen Pract. 1996; 2(2): 65–70. DOI: 10.3109/13814789609162146

[3] Bell C, Prior A, Frølich A, Appel CW, Vedsted P. Trajectories in Outpatient Care for People with Multimorbidity: A Population-Based Register Study in Denmark. Clin Epidemiol. 2022; 14: 749–62. DOI: 10.2147/CLEP.S363654 35686026 PMC9172733

[4] Freid VM, Bernstein AB, Bush MA. Multiple chronic conditions among adults aged 45 and over: trends over the past 10 years. NCHS Data Brief. 2012; 100: 1–8. 23101759

[5] Pefoyo AJ, Bronskill SE, Gruneir A, Calzavara A, Thavorn K, Petrosyan Y, et al. The increasing burden and complexity of multimorbidity. BMC public health. 2015; 15: 415-015-1733-2. DOI: 10.1186/s12889-015-1733-2 PMC4415224 25903064

[6] Wolff DL, Von Plessen C, Waldorff FB, Sorensen TL, Bogh SB, Rubin KH, et al. Time trends in patients managed simultaneously in multiple hospital outpatient specialty clinics for chronic diseases: A register-based cross-sectional study. J Comorb. 2019; 9: 2235042X19831907. DOI: 10.1177/2235042X19831907 PMC6416988 30891430

[7] Damarell RA, Morgan DD, Tieman JJ. General practitioner strategies for managing patients with multimorbidity: a systematic review and thematic synthesis of qualitative research. BMC Fam Pract. 2020; 21(1): 131. DOI: 10.1186/s12875-020-01197-8 32611391 PMC7331183

[8] Moth G, Vestergaard M, Vedsted P. Chronic care management in Danish general practice—a cross-sectional study of workload and multimorbidity. BMC Fam Pract. 2012; 13: 52-2296-13-52. DOI: 10.1186/1471-2296-13-52 PMC3436724 22676446

[9] Barnett ML, Bitton A, Souza J, Landon BE. Trends in Outpatient Care for Medicare Beneficiaries and Implications for Primary Care, 2000 to 2019. Ann Intern Med; 2021. DOI: 10.7326/M21-1523 PMC8688292 34724406

[10] Cassell A, Edwards D, Harshfield A, Rhodes K, Brimicombe J, Payne R, et al. The epidemiology of multimorbidity in primary care: a retrospective cohort study. Br J Gen Pract. 2018; 68(669): e245–e51. DOI: 10.3399/bjgp18X695465 29530918 PMC5863678

[11] Bayliss EA, Edwards AE, Steiner JF, Main DS. Processes of care desired by elderly patients with multimorbidities. Family practice. 2008; 25(4): 287–93. DOI: 10.1093/fampra/cmn040 18628243 PMC2504745

[12] Pedersen AF, Noroxe KB, Vedsted P. Influence of patient multimorbidity on GP burnout: a survey and register-based study in Danish general practice. Br J Gen Pract. 2020; 70(691): e95–e101. DOI: 10.3399/bjgp20X707837 31932298 PMC6960003

[13] Nissen NK, Aarhus R, Ørtenblad L. Dynamics of a specialized and complex health care system: Exploring general practitioners’ management of multimorbidity. Chronic Illn. 2020; 1742395320928403. DOI: 10.1177/1742395320928403 32498609

[14] Schmidt M, Schmidt SAJ, Adelborg K, Sundbøll J, Laugesen K, Ehrenstein V, et al. The Danish health care system and epidemiological research: from health care contacts to database records. Clin Epidemiol. 2019; 11: 563–91. DOI: 10.2147/CLEP.S179083 31372058 PMC6634267

[15] Hughes LD, McMurdo ME, Guthrie B. Guidelines for people not for diseases: the challenges of applying UK clinical guidelines to people with multimorbidity. Age Ageing. 2013; 42(1): 62–9. DOI: 10.1093/ageing/afs100 22910303

[16] Moffat K, Mercer SW. Challenges of managing people with multimorbidity in today’s healthcare systems. BMC Family Practice. 2015; 16: 129-015-0344-4. DOI: 10.1186/s12875-015-0344-4 PMC4604728 26462820

[17] Schiotz ML, Host D, Frolich A. Involving patients with multimorbidity in service planning: perspectives on continuity and care coordination. J Comorb. 2016; 6(2): 95–102. DOI: 10.15256/joc.2016.6.81 29090180 PMC5556451

[18] Gustafsson M, Kristensson J, Holst G, Willman A, Bohman D. Case managers for older persons with multi-morbidity and their everyday work – a focused ethnography. BMC Health Services Research. 2013; 13: 496-6963-13-496. DOI: 10.1186/1472-6963-13-496 PMC3893533 24279695

[19] Colombo F, Garcia-Goni M, Schwierz C. Addressing multimorbidity to improve healthcare and economic sustainability. J Comorb. 2016; 6(1): 21–7. DOI: 10.15256/joc.2016.6.74 29090168 PMC5556464

[20] Vedsted P, Olesen F. The Chronic Care Model and Risk Stratification [in Danish]. Månedsskr Prakt Lægegern. 2006; 84(April): 357–68.

[21] Wagner EH, Austin BT, Davis C, Hindmarsh M, Schaefer J, Bonomi A. Improving chronic illness care: translating evidence into action. Health Aff (Millwood). 2001; 20(6): 64–78. DOI: 10.1377/hlthaff.20.6.64 11816692

[22] World Health Organization. WHO global strategy on people-centred and integrated health services: interim report; 2015.

[23] Xu X, Mishra GD, Jones M. Evidence on multimorbidity from definition to intervention: An overview of systematic reviews. Ageing Res Rev. 2017; 37: 53–68. DOI: 10.1016/j.arr.2017.05.003 28511964

[24] Smith SM, Wallace E, O’Dowd T, Fortin M. Interventions for improving outcomes in patients with multimorbidity in primary care and community settings. Cochrane Database Syst Rev. 2021; 1: Cd006560. DOI: 10.1002/14651858.CD006560.pub4 33448337 PMC8092473

[25] Eriksen CU BH, Helding SAL, Frølich A. Organisational and patient-oriented interventions in multimorbidity – A systematic literature study of national and international experiences with organising health care for people with multimorbidity [in Danish]; 2019.

[26] van der Heide I, Snoeijs S, Quattrini S, Struckmann V, Hujala A, Schellevis F, et al. Patient-centeredness of integrated care programs for people with multimorbidity. Results from the European ICARE4EU project. Health Policy. 2018; 122(1): 36–43. DOI: 10.1016/j.healthpol.2017.10.005 29129270

[27] de Bruin SR, Versnel N, Lemmens LC, Molema CC, Schellevis FG, Nijpels G, et al. Comprehensive care programs for patients with multiple chronic conditions: a systematic literature review. Health Policy. 2012; 107(2–3): 108–45. DOI: 10.1016/j.healthpol.2012.06.006 22884086

[28] Hopman P, de Bruin SR, Forjaz MJ, Rodriguez-Blazquez C, Tonnara G, Lemmens LC, et al. Effectiveness of comprehensive care programs for patients with multiple chronic conditions or frailty: A systematic literature review. Health Policy. 2016; 120(7): 818–32. DOI: 10.1016/j.healthpol.2016.04.002 27114104

[29] Baxter S, Johnson M, Chambers D, Sutton A, Goyder E, Booth A. The effects of integrated care: a systematic review of UK and international evidence. BMC Health Serv Res. 2018; 18(1): 350. DOI: 10.1186/s12913-018-3161-3 29747651 PMC5946491

[30] Smith SM, Soubhi H, Fortin M, Hudon C, O’Dowd T. Managing patients with multimorbidity: systematic review of interventions in primary care and community settings. BMJ. 2012; 345: e5205. DOI: 10.1136/bmj.e5205 22945950 PMC3432635

[31] Lewis C, Wallace E, Kyne L, Cullen W, Smith SM. Training doctors to manage patients with multimorbidity: a systematic review. J Comorb. 2016; 6(2): 85–94. DOI: 10.15256/joc.2016.6.87 29090179 PMC5556450

[32] Rijken M, Hujala A, van Ginneken E, Melchiorre MG, Groenewegen P, Schellevis F. Managing multimorbidity: Profiles of integrated care approaches targeting people with multiple chronic conditions in Europe. Health Policy. 2018; 122(1): 44–52. DOI: 10.1016/j.healthpol.2017.10.002 29102089

[33] Ware J, Jr., Kosinski M, Keller SD. A 12-Item Short-Form Health Survey: construction of scales and preliminary tests of reliability and validity. Med Care. 1996; 34(3): 220–33. DOI: 10.1097/00005650-199603000-00003 8628042

[34] Salisbury C, Man MS, Bower P, Guthrie B, Chaplin K, Gaunt DM, et al. Management of multimorbidity using a patient-centred care model: a pragmatic cluster-randomised trial of the 3D approach. Lancet. 2018; 392(10141): 41–50. DOI: 10.1016/S0140-6736(18)31308-4 29961638 PMC6041724

[35] Damery S, Flanagan S, Combes G. Does integrated care reduce hospital activity for patients with chronic diseases? An umbrella review of systematic reviews. BMJ Open. 2016; 6(11): e011952. DOI: 10.1136/bmjopen-2016-011952 PMC5129137 27872113

[36] Flanagan S, Damery S, Combes G. The effectiveness of integrated care interventions in improving patient quality of life (QoL) for patients with chronic conditions. An overview of the systematic review evidence. Health Qual Life Outcomes. 2017; 15(1): 188. DOI: 10.1186/s12955-017-0765-y 28962570 PMC5622519

[37] Czypionka T, Kraus M, Reiss M, Baltaxe E, Roca J, Ruths S, et al. The patient at the centre: evidence from 17 European integrated care programmes for persons with complex needs. BMC Health Serv Res. 2020; 20(1): 1102. DOI: 10.1186/s12913-020-05917-9 33256723 PMC7706259

[38] Bell CPA, Frølich A, Appel CW, Vedsted P. Improving health care for patients with multimorbidity: A mixed-methods study to explore the feasibility and process of aligning scheduled outpatient appointments through collaboration between medical specialties. Int J Integr Care. 2022; 22(1): 17, 1–5. DOI: 10.5334/ijic.6013 PMC8896239 35340347

[39] Salisbury C, Johnson L, Purdy S, Valderas JM, Montgomery AA. Epidemiology and impact of multimorbidity in primary care: a retrospective cohort study. The British journal of general practice: the journal of the Royal College of General Practitioners. 2011; 61(582): e12–21. DOI: 10.3399/bjgp11X548929 21401985 PMC3020068

[40] Sinnott C, Mc Hugh S, Browne J, Bradley C. GPs’ perspectives on the management of patients with multimorbidity: systematic review and synthesis of qualitative research. BMJ Open. 2013; 3(9): e003610-2013. DOI: 10.1136/bmjopen-2013-003610 PMC3773648 24038011

[41] Prior A, Vestergaard CH, Ribe AR, Sandbæk A, Bro F, Vedsted P, et al. Chronic care services and variation between Danish general practices: a nationwide cohort study. British Journal of General Practice. 2021; 0419. DOI: 10.3399/BJGP.2021.0419 PMC8843375 34990398

[42] Larsen FB, PM, Lasgaard M. How are you? 2017 – Health profile for region and municipalties DEFAKTUM; 2018.

[43] Marengoni A, Vetrano DL, Calderon-Larranaga A, Onder G. Multimorbidity and patient-centred care in the 3D trial. Lancet. 2019; 393(10167): 127–8. DOI: 10.1016/S0140-6736(18)32528-5 30638574

[44] Birke H, Jacobsen R, Jønsson AB, Guassora ADK, Walther M, Saxild T, et al. A complex intervention for multimorbidity in primary care: A feasibility study. J Comorb. 2020; 10: 2235042x20935312. DOI: 10.1177/2235042X20935312 PMC7418232 32844099

[45] Manning E, Gagnon M. The complex patient: A concept clarification. Nursing and Health Sciences. 2017; 19: 13–21. DOI: 10.1111/nhs.12320 28054430

